# On the use of growth models to understand epidemic outbreaks with application to COVID-19 data

**DOI:** 10.1371/journal.pone.0240578

**Published:** 2020-10-20

**Authors:** Chénangnon Frédéric Tovissodé, Bruno Enagnon Lokonon, Romain Glèlè Kakaï

**Affiliations:** Laboratoire de Biomathématiques et d’Estimations Forestières, Faculté des Sciences Agronomiques, Université d’Abomey-Calavi, Abomey-Calavi, Bénin; Universita degli Studi di Catania, ITALY

## Abstract

The initial phase dynamics of an epidemic without containment measures is commonly well modelled using exponential growth models. However, in the presence of containment measures, the exponential model becomes less appropriate. Under the implementation of an isolation measure for detected infectives, we propose to model epidemic dynamics by fitting a flexible growth model curve to reported positive cases, and to infer the overall epidemic dynamics by introducing information on the detection/testing effort and recovery and death rates. The resulting modelling approach is close to the Susceptible-Infectious-Quarantined-Recovered model framework. We focused on predicting the peaks (time and size) in positive cases, active cases and new infections. We applied the approach to data from the COVID-19 outbreak in Italy. Fits on limited data before the observed peaks illustrate the ability of the flexible growth model to approach the estimates from the whole data.

## Introduction

COVID-19 is a pandemic caused by the new coronavirus strain SARS-nCOV2 which emerged from Wuhan, China [1, 2]. A total of 21 026 758 COVID-19 cases and 755 786 related deaths were reported across the world as at August 15, 2020 [3]. The worldwide social, as well as economic ravages by COVID-19 has immediately motivated the use of mathematical models to understand the course of the epidemic and plan for effective control strategies. These include, for instance, the SIR (Susceptible, Infectious, Recovered), SEIR (Susceptible, Exposed, Infectious, Recovered) and its variants, SIDR (Susceptible, Infectious, Recovered, Dead) and SIQR (Susceptible, Infectious, Quarantined, Recovered) models [4–7]. These modelling approaches use mechanistic models which incorporate key physical laws or mechanisms involved in the dynamics of the population at risk and the pathogen [8]. A second class of approaches uses empirical phenomenological models which does not require specific knowledge on the physical laws or mechanisms that give rise to the observed epidemic data [9], and was considered, for instance, by [10] and [11] to understand both short and long term dynamics of COVID-19. A new curve fitting-like approach, namely fractal interpolation [12, 13] was also proposed by [14–16] to account for the high noise and reporting bias in data from the COVID-19 pandemic. As generally is the case with dynamic biological systems [17, 18], mathematical model development and adaptation are fundamental requirements to guide public health policies.

When facing an epidemic outbreak, public health officials are mostly interested in data driven, mathematically motivated, practical and computationally efficient approaches that can: *i*) generate estimates of key transmission parameters; *ii*) gain insight to the contribution of different transmission pathways; *iii*) assess the impact of control interventions (*e.g*. social distancing, test + isolation, vaccination campaigns); *iv*) optimize the impact of control strategies; and *v*) generate short and long-term forecasts [8]. In regard to the current COVID-19 outbreak, politics and public health officials are mostly worried about the ability of the disease to induce saturation of the health system, reducing the survival of patients, and even consulting for reasons different from the epidemic itself. High interest is thus currently given to accurate forecasting of the epidemic peak time and size, epidemic size and duration, as well as their sensitivity to control interventions in order to optimize the impact of control strategies.

An exponential-growth model is usually assumed to characterize the early phase of epidemics. But, this assumption can lead to failure to appropriately capture the profile of the epidemic growth, eventually giving rise to non-realistic epidemic forecasts [10, 19]. In an ultimate view to guide control interventions aiming to limit the spread of epidemics, with focus on the COVID-19 pandemic, this work considered a flexible growth curve fitting approach to understand the dynamics of epidemics. We used the generic growth model of [20] to model the course of reported positive cases and a binomial regression to model removals (recoveries and deaths). Thereafter, we inferred the overall dynamics of the epidemic, in terms of observables (reported cases, active/quarantined cases) and unobservables (new infections, lost cases), and predicted interest quantities such as the peak (time and size) in reported cases, active cases and new infections. The performance of the approach was assessed through an application to daily case reporting data from Italy, which has virtually completed a whole COVID-19 outbreak wave, thus offering the possibility to compare predicted outputs to real events.

## Methods

We used a growth curve approach for modeling the course of an epidemic along time. We followed [8, 10, 21] who, among others, used growth models to forecast epidemic dynamics.

### Structural model for epidemic incidence

Let *C*_*t*_ denote the size of the detected infected population at time *t*, *i.e*. the cumulative number of infected, identified and isolated individuals. We assumed for convenience that *C*_*t*_ is continuous and denote ${\dot{C}}_{t}$ its first derivative with respect to *t*. Also let *I*_*t*_ be the true size of infectives at *t*, related to *C*_*t*_ through

$$\begin{array}{c} {\dot{C}}_{t}=\delta_{t}I_{t} \end{array}$$ (1)

where *δ*_*t*_ ∈ (0, 1] is the detection rate which is closely related to the testing effort (number of tests, tracing of contact persons of identified cases and targeting exposed people) and is assumed at least twice differentiable with respect to *t*. We ressorted to the generic growth model of [20] for the identified positive cases:

$$\begin{array}{c} C_{t}=K{\left(1+u_{t}\right)}^{-1/\nu } \end{array}$$ (2)

with *u*_*t*_ = [1 + *νωρ*(*t* − *τ*)]^−1/*ρ*^. In Eq (2), *K* > 0 is the ultimate epidemic size (detected), *ω* > 0 is the “intrinsic” growth constant, *ν* and *ρ* are powers (*ν* > 0 and −1 < *ρ* < *ν*^−1^) characterizing respectively the rates of change with respect to the initial size *C*_0_ = *δ*_0_ *I*_0_ (number of cases detected at time *t* = 0) and the ultimate size *K*, and *τ* is a constant of integration, determined by the initial conditions of the epidemic and implicitly the detection rate *δ*_0_ through *C*_0_ = *K*[1 + (1 − *νωρτ*)^−1/*ρ*^]^−1/*ν*^ for *ρ* ≠ 0 and *C*_0_ = *K*(1 + *e*^*νωτ*^)^−1/*ν*^ for *ρ* = 0. The growth model in Eq (2) is quite flexible to handle various shapes of epidemic dynamics. Indeed, if *K* → ∞ and *νρ* → 0, Eq (2) specializes to the exponential growth model

$$\begin{array}{c} C_{t}={\text{e}}^{\omega (t-\tau )} \end{array}$$ (3)

where *ω* is the exponential growth rate. Apart from Eq (3), other special or limiting cases of Eq (2) include the hyper-Gompertz (*ν* → 0 while *ων*^1+*ρ*^ is constant) and the Gompertz (*ν* → 0, *ρ* → 0 while *ων* is constant), the Bertalanffy-Richards (*ρ* → 0), the hyper-logistic (*ν* = 1) and the logistic (*ν* = 1 and *ρ* → 0) growth models [20]. From Eq (2), the observed epidemic incidence ${\dot{C}}_{t}$ is given by

$$\begin{array}{c} {\dot{C}}_{t}=K\omega u_{t}^{1+\rho }{\left(1+u_{t}\right)}^{-\frac{\nu +1}{\nu }}. \end{array}$$ (4)

In order to ensure the restriction −1 < *ρ* < *ν*^−1^, we set $\rho =\rho_{0}\frac{\nu +1}{\nu }-1$ with *ρ*_0_ ∈ (0, 1) free of *ν*.

### Active cases and outcomes

The number *A*_*t*_ of detected and active cases along an epidemic outbreak is of high interest for public health officials. Indeed, *A*_*t*_ must be kept under the carrying capacity of the health system to avoid overload and disrupture. The derivative ${\dot{A}}_{t}$ of the detected and active cases satisfies

$$\begin{array}{ccc} {\dot{A}}_{t}\;\; & = & \;\;{\dot{C}}_{t}-R_{t} \end{array}$$ (5)

where *R*_*t*_ = *α*_*t*_ *A*_*t*_ denotes the number of removed and permanently immune (mortality and recovery) at time *t*, and *α*_*t*_ is the unit time removal probability, *i.e*. the odds to have an outcome (recovery or death), averaged over the active cases. Eq (5) fits in the SIQR (Susceptible, Infectious, Quarantined, Recovered) model framework [22] with the detected active cases referred to as “quarantined” and the strong assumption that *α*_*t*_ is constant along the epidemic outbreak (see the third equation in system (6) in [22]). The removal probability can more generally be given the logistic form $\alpha_{t}=\frac{{\text{e}}^{\eta_{t}}}{1+{\text{e}}^{\eta_{t}}}$ with $\eta_{t}=X_{t}^{⊤}\;\beta +\kappa t$ where ***X***_*t*_ = (*X*_*t*1_, *X*_*t*2_, ⋯, *X*_*tq*_)^⊤^ is a vector of *q* covariates (known constants) and ***β*** is the *q* vector of associated effects, and *κ* determines the change in the log-odds ratio for having an outcome per unit time. These changes in *α*_*t*_ can be due to an improvement in the health care system during the epidemic outbreak (increase in recovery ratio) or a deterioration of the health care system for infected individuals (increase in mortality ratio due to the outbreak). The general solution of the differential Eq (5) turns to have the form

$$\begin{array}{ccc} A_{t}\;\; & = & \;\;\{\begin{array}{cc} \;\;\;\;\;\;\;\;\;\;[A_{0}+\int_{0}^{t}{\dot{C}}_{s}{\text{e}}^{\alpha_{s}s}ds]{\text{e}}^{-\alpha_{t}t}\;\;\;\;\;\;\;\;\;\;\;\;\;\;\;\;\;\;\;\;\;\;\;\;\;\;\;\;\;\;\;\;\; & \;\text{if}\;\;\;\kappa =0\\ {}[A_{0}\;{\left(1+{\text{e}}^{X_{t}^{⊤}\;\beta }\right)}^{1/\kappa }+\int_{0}^{t}{\dot{C}}_{s}{\left(1+{\text{e}}^{\eta_{s}}\right)}^{1/\kappa }ds]{\left(1+{\text{e}}^{\eta_{t}}\right)}^{-1/\kappa }\; & \;\text{if}\;\;\;\kappa \neq 0 \end{array} \end{array}$$ (6)

where *A*_0_ is the number of active cases at time *t* = 0. Indeed, when *κ* = 0, taking the first derivative of *A*_*t*_ yields ${\dot{A}}_{t}={\dot{C}}_{t}{\text{e}}^{\alpha_{t}t}{\text{e}}^{-\alpha_{t}t}+[A_{0}+\int_{0}^{t}{\dot{C}}_{s}{\text{e}}^{\alpha_{s}s}ds](-\alpha_{t}){\text{e}}^{-\alpha_{t}t}$ resulting in ${\dot{A}}_{t}={\dot{C}}_{t}-\alpha_{t}A_{t}$ which is the Eq (5). For *t* = 0, the integral in Eq (6) vanishes, resulting as expected in *A*_*t*_ = *A*_0_ since ${\text{e}}^{-\alpha_{t}t=1}$. When *κ* ≠ 0, the first derivative of *A*_*t*_ is ${\dot{A}}_{t}={\dot{C}}_{t}{\left(1+{\text{e}}^{\eta_{t}}\right)}^{1/\kappa }{\left(1+{\text{e}}^{\eta_{t}}\right)}^{-1/\kappa }+A_{t}(\frac{1}{-\kappa })(\kappa {\text{e}}^{\eta_{t}}){\left(1+{\text{e}}^{\eta_{t}}\right)}^{-1}={\dot{C}}_{t}-\frac{{\text{e}}^{\eta_{t}}}{1+{\text{e}}^{\eta_{t}}}A_{t}$ which reduces to ${\dot{A}}_{t}={\dot{C}}_{t}-\alpha_{t}A_{t}$ in accordance with Eq (5). Here, for *t* = 0, ${\text{e}}^{\eta_{t}}={\text{e}}^{X_{t}^{⊤}\;\beta }$ so that *A*_*t*_ = *A*_0_.

There are no general closed form solutions for the integrals in Eq (6), unless ${\dot{C}}_{t}$ and *α*_*t*_ are purposely chosen as functions of time to simplify the integral. *A*_*t*_ can, however, be obtained in practice from Eq (6) using a numerical integration routine such as the function *integrate* in R freeware [23] or the function *integral* of Matlab [24]. Nevertheless, to circumvent this issue during estimation under the generic growth model in Eq (2), we discretized the active cases *A*_*t*_ by assuming a binomial removal process *R*_*t*_ conditional on the detected unit time new cases *Y*_*t*_ as

$$\begin{array}{ccc} R_{t}|A_{t-1},Y_{t}\;\; & ∼ & \;\;\text{BIN}(A_{t-1}+Y_{t},\alpha_{t}) \end{array}$$ (7)

$$\begin{array}{ccc} A_{t} & = & A_{t-1}+Y_{t}-R_{t} \end{array}$$ (8)

where BIN(*n*, *α*) denotes a binomial distribution with *n* trials and success probability *α* and *Y*_*t*_ is a non-negative process with expectation $\lambda_{t}={\dot{C}}_{t}$. Clearly, the bivariate process {*A*_*t*_, *R*_*t*_} defined by Eqs (8) and (7) is not stationary. However, since *Y*_*t*_ ≥ 0 and ${\dot{C}}_{t}\to 0$ as *t* → ∞, we have *Y*_*t*_ → 0 in distribution as *t* → ∞, and if the removal probability *α*_*t*_ does not approach zero as *t* → ∞, then *A*_*t*_ → 0 as *t* → ∞.

### Peak of detected cases

The epidemic peak is an important event in the disease dynamic and can be estimated for a better management of the epidemic. An epidemic described by the exponential growth model in Eq (3), (*K* → ∞ or *νρ* → 0) does not peak. Otherwise, the peak in the detected number of infected individuals corresponds to the maximum of the incidence rate ${\dot{C}}_{t}$. This maximum is then attained when ${\ddot{C}}_{t}=\frac{\partial {\dot{C}}_{t}}{\partial t}=0$. We have from Eq (4)

$$\begin{array}{ccc} {\ddot{C}}_{t} & = & \nu \omega u_{t}^{\rho }[\frac{\nu +1}{\nu }\frac{u_{t}}{1+u_{t}}-(1+\rho )]{\dot{C}}_{t}. \end{array}$$ (9)

Solving ${\ddot{C}}_{t}=0$ for *t* using Eq (9) yields the peak time $t_{p}=\tau +\{{\left[\frac{1-\nu \rho }{\nu (1+\rho )}\right]}^{\rho }-1\}/\left(\nu \omega \rho \right)$ which reads,

$$\begin{array}{c} t_{p}=\tau +\frac{1}{\omega [\rho_{0}-\left(1-\rho_{0}\right)\nu ]}[{\left(\frac{\rho_{0}}{1-\rho_{0}}\right)}^{1-\rho_{0}\left(\nu +1\right)/\nu }-1] \end{array}$$ (10)

on replacing $\rho =\rho_{0}\frac{\nu +1}{\nu }-1$. Inserting *t*_*p*_ in Eq (1) and denoting $u_{p}=\nu \frac{1+\rho }{1-\rho \nu }$ gives the peak

$$\begin{array}{c} {\dot{C}}_{p}=K\omega u_{p}^{1+\rho }{\left(\frac{\nu +1}{1-\rho \nu }\right)}^{-\frac{\nu +1}{\nu }}. \end{array}$$ (11)

At the peak in detected cases, the cumulative number of detected cases is *C*_*p*_ = *K*(1 + *u*_*p*_)^−1/*ν*^.

### Overall epidemic dynamics

An important interest in modelling the epidemic incidence is the derivation of quantities related to the overall dynamics of the epidemic, in both detected and undetected cases.

#### Total cases: Detected and losts

Let us denote *S*_*t*_ the cumulative number of cases from the epidemic outbreak to *t*, and let ${\dot{S}}_{t}$ be the first derivative of *S*_*t*_. We also introduce Λ_*t*_, the cumulative number of lost cases (with first derivative ${\dot{\Lambda }}_{t}$), *i.e*. people who were infected, undetected, and removed from infectives (mortality and recovery).

The size of the lost cases is determined by the unit time removal rate *π*_*t*_ ∈ (0, 1) from undetected infectives (*π*_*t*_ is an average over all infectives, *i.e*. irrespective of the time since infection onset). The lost rate *π*_*t*_ which is assumed at least twice differentiable with respect to *t*, depends on various factors like the disease related mortality, the average infection duration, the natural proportion of asymptomatics within infectives, and the existence and the use of medicines that may reduce symptoms (induced asymptomatics). It is worthwhile noticing that *π*_*t*_ can be estimated from the removal rate *α*_*t*_ in the detected cases, taking into account various factors that may induce difference between the two rates. For instance, since the undetected cases include asymptomatics, disease related mortality may be lower and recovery rate higher in undetected as compared to detected cases. However, efficiency of the health care system in treating identified and isolated cases can reduce mortality thereby reducing *α*_*t*_, but also improve recovery thereby increasing *α*_*t*_.

With the above notations, the lost cases count Λ_*t*_ satisfies the differential equation,

$$\begin{array}{c} {\dot{\Lambda }}_{t}=\pi_{t}\left(1-\delta_{t}\right)I_{t} \end{array}$$ (12)

whereas the cumulative number of cases *S*_*t*_ is given on setting *υ*_*t*_ = (1 − *π*_*t*_)(1 − *δ*_*t*_) by

$$\begin{array}{c} S_{t}=C_{t}+\Lambda_{t}+\upsilon_{t}I_{t}. \end{array}$$ (13)

The factor *υ*_*t*_ represents at time *t* the proportion of infectives who will potentially continue to spread the epidemic after adequate contacts (*i.e*. contacts sufficient for transmission) with susceptibles. In other words, the number of undetected currently infectives is $\left(1-\pi_{t}\right)\left(\delta_{t}^{-1}-1\right){\dot{C}}_{t}$. From Eq (1), the infectives *I*_*t*_ and its first derivative with respect to time ${\dot{I}}_{t}$ are given for *t* ≥ 0 by

$$\begin{array}{ccc} I_{t} & = & \delta_{t}^{-1}{\dot{C}}_{t} \end{array}$$ (14)

$$\begin{array}{ccc} {\dot{I}}_{t} & = & \delta_{t}^{-1}[{\ddot{C}}_{t}-{\dot{\delta }}_{t}\delta_{t}^{-1}{\dot{C}}_{t}] \end{array}$$ (15)

where ${\dot{\delta }}_{t}$ is the first derivative of the detection rate *δ*_*t*_ with respect to *t*. Straightforward algebraic operations then give the number of new cases and the cumulative number of cases as

$$\begin{array}{ccc} {\dot{S}}_{t} & = & [\pi_{t}\delta_{t}^{-1}+(1-\pi_{t})(1-(\delta_{t}^{-1}-1)\delta_{t}^{-1}{\dot{\delta }}_{t})+{\dot{\upsilon }}_{t}\delta_{t}^{-1}]\;{\dot{C}}_{t}\\ & & +(1-\pi_{t})(\delta_{t}^{-1}-1){\ddot{C}}_{t} \end{array}$$ (16)

$$\begin{array}{ccc} S_{t} & = & C_{t}+\Lambda_{t}+(1-\pi_{t})(\delta_{t}^{-1}-1){\dot{C}}_{t} \end{array}$$ (17)

where ${\dot{\upsilon }}_{t}=-(1-\pi_{t}){\dot{\delta }}_{t}-(1-\delta_{t}){\dot{\pi }}_{t}$ with ${\dot{\pi }}_{t}$ the first derivative of the lost rate *π*_*t*_, and the cumulative number of lost cases Λ_*t*_ is given for *t* ≥ 0 by

$$\begin{array}{c} \Lambda_{t}=S_{0}+\int_{0}^{t}\pi_{s}\left(\delta_{s}^{-1}-1\right){\dot{C}}_{s}ds \end{array}$$ (18)

with *S*_0_ the cumulative number of all cases until the first detection date *t* = 0. The total size of the epidemic is *S*_∞_ = *C*_∞_ + Λ_∞_ since ${\dot{C}}_{t}\to 0$ as *t* → ∞. Under the Turner’s growth model, *S*_∞_ = *K* + Λ_∞_.

Let us assume a constant detection rate *δ*_*t*_ = *δ* closely related to detection effort but also to the average duration from infection to recovery or death of non-isolated cases. Assuming in addition a constant lost rate (*π*_*t*_ = *π*), we have ${\dot{\delta }}_{t}={\dot{\pi }}_{t}={\dot{\upsilon }}_{t}=0$, and the new cases ${\dot{S}}_{t}$ and its accumulation *S*_*t*_, as well as the lost cases Λ_*t*_ simplify to

$$\begin{array}{ccc} {\dot{S}}_{t} & = & [1+\pi (\delta^{-1}-1)]{\dot{C}}_{t}+(1-\pi )(\delta^{-1}-1){\ddot{C}}_{t} \end{array}$$ (19)

$$\begin{array}{ccc} S_{t} & = & S_{0}+[1+\pi (\delta^{-1}-1)]C_{t}+(1-\pi )(\delta^{-1}-1){\dot{C}}_{t} \end{array}$$ (20)

$$\begin{array}{ccc} \Lambda_{t} & = & S_{0}+\pi \left(\delta^{-1}-1\right)C_{t}. \end{array}$$ (21)

The total epidemic size is here *S*_∞_ = *S*_0_ + [1 + *π*(*δ*^−1^ − 1)]*K*.

#### Epidemic peak

At the time *t*_*p*_ of the peak of reported cases (${\ddot{C}}_{t}=0$) under constant detection and lost rates, the new infectives is ${\dot{S}}_{p}=[1+\pi (\delta^{-1}-1)]{\dot{C}}_{p}$ with ${\dot{C}}_{p}$ given in Eq (11). This, however, corresponds to the peak in the overall new cases ${\dot{S}}_{t}$ only under the unrealistic assumption *δ* = 1. The peak of new infections occurs when the second derivative ${\ddot{S}}_{t}$ of *S*_*t*_ with respect to *t* vanishes (${\ddot{S}}_{t}=0$). We have from Eq (16)

$$\begin{array}{ccc} {\ddot{S}}_{t} & = & [\pi_{t}\delta_{t}^{-1}+(1-\pi_{t})(1-(\delta_{t}^{-1}-1)\delta_{t}^{-1}{\dot{\delta }}_{t})+{\dot{\upsilon }}_{t}\delta_{t}^{-1}]{\ddot{C}}_{t}\\ & & +(1-\pi_{t})(\delta_{t}^{-1}-1){\dddot{C}}_{t}+\Psi_{t} \end{array}$$ (22)

where ${\dddot{C}}_{t}$ (the third derivative of *C*_*t*_ with respect to *t*) and Ψ_*t*_ are given by

$$\begin{array}{ccc} {\dddot{C}}_{t} & = & \nu^{2}\omega^{2}u_{t}^{2\rho }[\left(1+\rho \right)\left(2\rho +1\right)-\frac{3(\nu +1)(\rho +1)}{\nu }z_{t}\\ & & \;\;\;\;\;\;\;\;\;\;\;\;+\frac{\left(\nu +1)(2\nu +\nu \rho +1\right)}{\nu^{2}}z_{t}^{2}]{\dot{C}}_{t} \end{array}$$ (23)

$$\begin{array}{ccc} \Psi_{t} & = & \{{\dot{\pi }}_{t}[\delta_{t}^{-1}(1+(\delta_{t}^{-1}-1){\dot{\delta }}_{t})-1]+(1-\pi_{t})\delta_{t}^{-1}[{\ddot{\upsilon }}_{t}-(\delta_{t}^{-1}-1){\ddot{\delta }}_{t}]\\ & & +\delta_{t}^{-2}{\dot{\delta }}_{t}[(1-\pi_{t})[{\dot{\pi }}_{t}\left(1-\delta_{t}\right)+{\dot{\delta }}_{t}\left(2\delta_{t}^{-1}-\pi_{t}\right)]-\pi_{t}]\}{\dot{C}}_{t}\\ & & -[{\dot{\pi }}_{t}(\delta_{t}^{-1}-1)+(1-\pi_{t})\delta_{t}^{-2}{\dot{\delta }}_{t}]{\ddot{C}}_{t} \end{array}$$ (24)

with $z_{t}=\frac{u_{t}}{1+u_{t}}$, and ${\ddot{\pi }}_{t}$ and ${\ddot{\delta }}_{t}$ the second derivatives of respectively *π*_*t*_ and *δ*_*t*_ with respect to *t*. The peak time and value depend on the particular forms of *δ*_*t*_ and *π*_*t*_ as functions of time. Here, we restrict the attention to the simple situation with constant positive detection and lost rates (*δ*_*t*_ = *δ* with *δ* ∈ (0, 1) and *π*_*t*_ = *π* with *π* ∈ (0, 1)) where ${\dot{\delta }}_{t}={\dot{\pi }}_{t}=\Psi_{t}=0$ and Eq (22) reduces to

$$\begin{array}{ccc} {\ddot{S}}_{t} & = & [1+\pi (\delta^{-1}-1)]{\ddot{C}}_{t}+(1-\pi )(\delta^{-1}-1){\dddot{C}}_{t}. \end{array}$$ (25)

It appears that the peak of new infections occurs before the time *t*_*p*_ of the peak in detected cases. Indeed, at *t* = *t*_*p*_, we have ${\ddot{C}}_{t}=0$, (1 − *π*)(*δ*^−1^ − 1) > 0 and ${\dddot{C}}_{t}<0$ so that ${\ddot{S}}_{t}<0$, *i.e*. ${\dot{S}}_{t}$ is already in its descending phase. The expression Eq (25) indicates that at the time *t*_*P*_ of the peak of new infections, ${\ddot{C}}_{t}$ is equal to ${\ddot{C}}_{P}=-\zeta {\dddot{C}}_{P}$ where $\zeta =\frac{\left(1-\pi )(1-\delta \right)}{\pi +\delta (1-\pi )}$ and ${\dddot{C}}_{P}$ is given by Eq (23) with *t* = *t*_*P*_. The lower *ζ*, the lower $|{\ddot{C}}_{P}|$, and the lower the difference *t*_*p*_ − *t*_*P*_ (delay of the observed peak). Differentiating *ζ* with respect to *δ* gives $\frac{\partial \zeta }{\partial \delta }=-\frac{1-\pi }{{\left[\pi +\delta (1-\pi )\right]}^{2}}<0$, hence the higher *δ*, the lower the delay between the observed peak time and the time of the peak in new infections. Using Eqs (9) and (23), ${\ddot{S}}_{t}$ becomes

$$\begin{array}{ccc} {\ddot{S}}_{t} & = & \nu \omega u_{t}^{\rho }\delta^{-1}\{\frac{\nu \omega (1-\pi )(1-\delta )}{1+\nu \omega \rho (t-\tau )}[\left(1+\rho \right)\left(2\rho +1\right)-\frac{3(\nu +1)(\rho +1)}{\nu }z_{t}\\ & & \;\;\;\;\;\;\;\;\;\;\;\;\;\;\;\;\;\;\;\;\;\;\;\;\;\;\;\;\;\;\;\;\;\;\;\;\;\;\;\;+\frac{\left(\nu +1)(2\nu +\nu \rho +1\right)}{\nu^{2}}z_{t}^{2}]\\ & & \;\;\;\;\;\;\;\;\;\;\;\;\;+[\delta +\pi (1-\delta )][\frac{\nu +1}{\nu }z_{t}-\left(1+\rho \right)]\}{\dot{C}}_{t} \end{array}$$ (26)

which does not have a closed form root. The root *t*_*P*_ can, however, be obtained using root finding numerical routines such as the R function *uniroot* or the Matlab function *fzero*. Afterwards, the peak ${\dot{S}}_{p}$ size (the maximum number of new infections) is obtained using Eq (19).

### Statistical models and inference

Let us consider a record of new confirmed infected cases *Y*_1_, *Y*_2_, ⋯, *Y*_*n*_, active cases *A*_0_, *A*_1_, ⋯, *A*_*n*−1_, removed cases *R*_1_, *R*_2_, ⋯, *R*_*n*_ (available from Eq (8) as *R*_*t*_ = *Y*_*t*_ − *A*_*t*_ + *A*_*t*−1_) and the associated vectors of covariates *X*_1_, *X*_2_, ⋯, *X*_*n*_ at *n* time points. The parameters *K*, *ω*, *ν*, *ρ*_0_, *τ*, and *κ* can be estimated using maximum likelihood (ML) by assigning to each *Y*_*t*_ an appropriate statistical distribution with expectation $\lambda_{t}={\dot{C}}_{t}$ and a dispersion parameter *σ* > 0, and probability density function (pdf) or probability mass function (pmf) *f*(*Y*_*t*_|***θ***) where ***θ*** = (*K*, *ω*, *ν*, *ρ*_0_, *τ*, *κ*, *β*^⊤^, *σ*)^⊤^. We subsequently considered inference under log-normal and negative binomial distributions.

#### Log-normal model

Epidemic incidence case data are generally fitted through non-linear least squares applied at logarithmic scale [19, 25, 26]. To deal with zero incidence cases, the logarithmic transform is usually applied on the shifted cases *Y*_*t*_ + 1. Mimicking this procedure in a likelihood inference framework, we consider a log-normal distribution assumption for the shifted incidence cases, *i.e*. *Y*_*t*_ + 1 ∼ *LN*(λ_*t*_ + 1, *σ*). The pdf of *Y*_*t*_, adapted from [27], reads

$$\begin{array}{c} f\left(Y_{t}|\theta \right)=\frac{1}{\sigma \left(Y_{t}+1\right)\sqrt{2\pi }}\text{exp}\{-\frac{1}{2}{\left(\frac{\text{log}\left(Y_{t}+1\right)-\text{log}\left(\lambda_{t}+1\right)}{\sigma }+\frac{\sigma }{2}\right)}^{2}\} \end{array}$$ (27)

so that *Y*_*t*_ has expectation *E*[*Y*_*t*_] = λ_*t*_ and variance $Var\left[Y_{t}\right]={\left(\lambda_{t}+1\right)}^{2}({\text{e}}^{\sigma^{2}}-1)$.

#### Negative binomial model

Since incidence cases are counts, *Y*_*t*_ can be assumed to follow the negative binomial distribution, *i.e*. *Y*_*t*_ ∼ *NB*(λ_*t*_, *σ*) with pmf

$$\begin{array}{c} f\left(Y_{t}|\theta \right)=\frac{\Gamma (Y_{t}+1/\sigma )}{\Gamma \left(Y_{t}+1\right)\Gamma \left(1/\sigma \right)}{\left(\frac{\sigma \lambda_{t}}{\sigma \lambda_{t}+1}\right)}^{1/\sigma }{\left(\frac{1}{\sigma \lambda_{t}+1}\right)}^{Y_{t}}. \end{array}$$ (28)

The incidence case *Y*_*t*_ then has expectation *E*[*Y*_*t*_] = λ_*t*_ and variance *Var*[*Y*_*t*_] = λ_*t*_(1 + *σλ*_*t*_).

#### Likelihood inference

Based on the information {*Y*_*t*_, *R*_*t*_} for *t* = 1, 2, ⋯, *n*, the conditional log-likelihood of the parameter ***θ*** given *A*_0_ is

$$\begin{array}{c} \ell \left(\theta \right)=\sum_{t=1}^{n}[\text{log}\;f\left(Y_{t}|\theta \right)+\text{log}\;f_{B}\left(R_{t}|\theta \right)] \end{array}$$ (29)

where $f_{B}\left(R_{t}|\theta \right)=(\begin{array}{c} A_{t-1}+N_{t}\\ R_{t} \end{array})\alpha_{t}^{R_{t}}{\left(1-\alpha_{t}\right)}^{A_{t-1}+N_{t}-R_{t}}$ is the binomial probability mass function for *R*_*t*_. The function *ℓ*(⋅) can be maximized to obtain the maximum likelihood estimate $\hat{\theta }$ of ***θ*** using an optimization routine such as the function *optim* in R or the function *fminsearch* of Matlab. Let *H*(***θ***) the hessian matrix of *ℓ*(***θ***) and define the covariance Σ(*θ*) = −[*H*(*θ*)]^−1^. The large sample distribution (*i.e*. for *n* → ∞) of the maximum likelihood estimator is multivariate normal with mean $\hat{\theta }$ and covariance matrix $\hat{\Sigma }=\Sigma (\hat{\theta })$.

### Application to reported COVID-19 new cases in Italy

#### The data

In order to test the reliability of the Turner’s growth model in predicting the dynamics of an epidemic, we used data from one of the countries which had completed a whole COVID-19 outbreak wave. The daily case reporting data in Italy was obtained from https://github.com/CSSEGISandData/COVID-19/tree/master/csse_covid_19_data/csse_covid_19_time_series. We used only the confirmed data (2020-02-20 to 2020-07-11) accessed on 2020-07-28, discarding the latest data subject to possible reporting delay, as indicated by the Istituto Superiore di Sanità (ISS) at https://www.epicentro.iss.it/en/coronavirus/sars-cov-2-dashboard.

#### Data analysis

All analyses were performed in R [23]. We fitted the Turner’s growth model curve to the whole Italian data. Both the log-normal and the negative binomial distributions were used, and the fit with the lowest root mean square error (RMSE) computed for the daily new positive cases was selected as the best. Then, we derived peak statistics (time and size) for daily new reported cases and active cases. We also inferred the daily new infections from assuming constant detection and lost rates and estimated its peak (time and size). The detection (*δ* = 0.033/day) and the lost rates (*π* = 0.1/day) for Italy were obtained from [7]. These rates follow from assumptions that the average time of duration from infection to recovery or death of non-isolated cases is 10 days (hence *π* = 0.1/day) and that during this detection window, 1/3 of infectives are tested positives (hence *δ* = 0.033/day).

To assess the ability of the model in predicting the peak of the new positive cases in countries which have not yet reached the peak, we retrospectively fitted the model to the Italian data before the observed peak (day 29 after the notification of first case), using data of the first two weeks, and then data of the first three weeks. For these analyses with limited data, we fitted the full Turner’s growth model to the positive cases, but also its special cases, namely the hyper-Gompertz (*ν* → 0 while *ων*^1+*ρ*^ is constant), the Gompertz (*ν* → 0, *ρ* → 0 while *ων* is constant), the Bertalanffy-Richards (*ρ* → 0), the hyper-logistic (*ν* = 1) and the logistic (*ν* = 1 and *ρ* → 0) models using the log-normal distribution for the daily counts. We then computed the Akaike’s Information Criterion (AIC) defined as AIC $=-2\hat{\ell }+2N_{p}$ with $\hat{\ell }$ the maximized log-likelihood and *N*_*p*_ the number of parameters in a fitted model. Finally, we retained and presented the best fit (lowest AIC value).

## Results

### Modelling the whole Italian data

**Fig 1:**
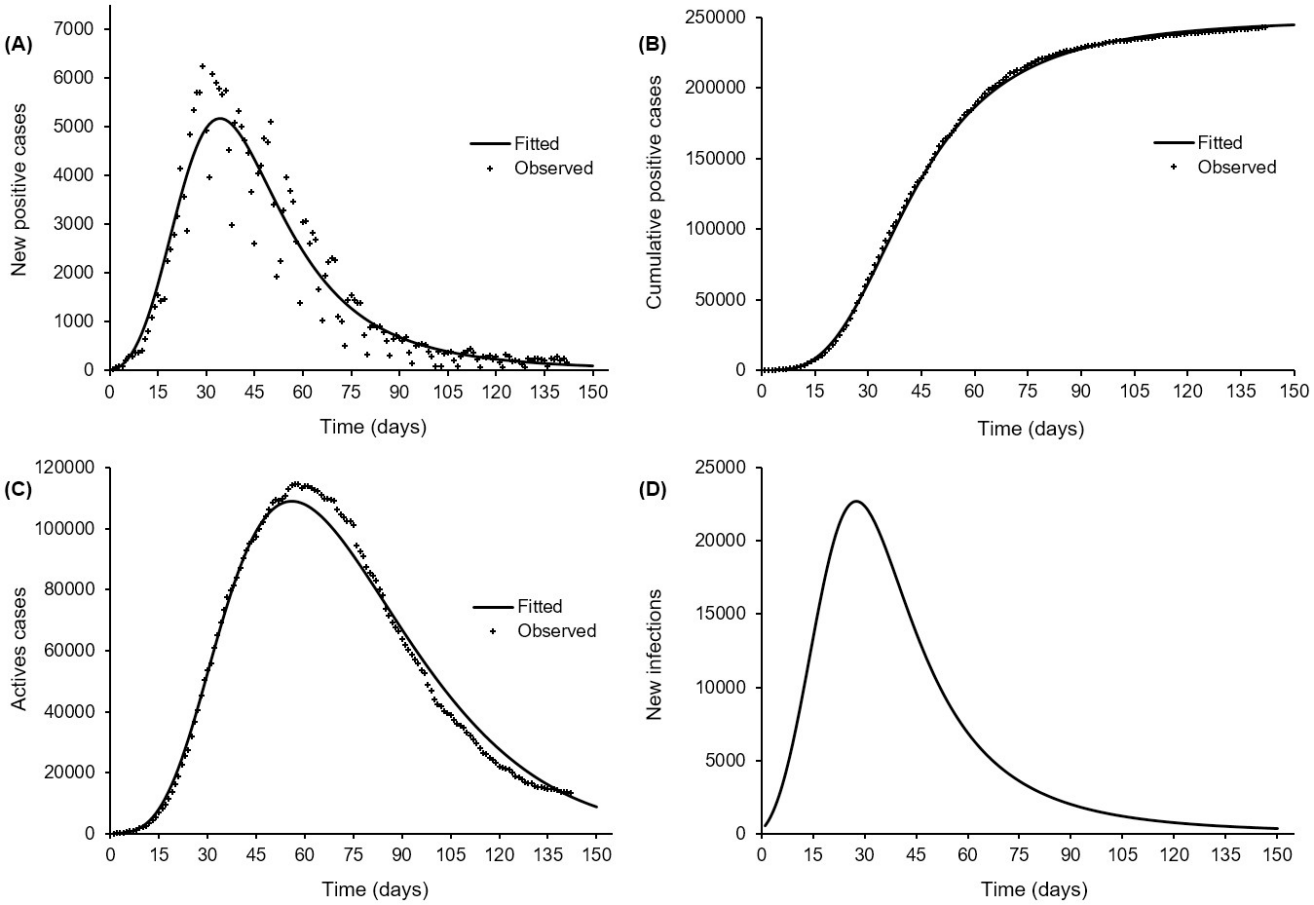
Log-normal fit of Turner’s model to the COVID-19 daily case reporting data from Italy (2020-02-20 to 2020-07-11). New reported cases (A), cumulative positive cases (B), active (quarantined) cases (C) and estimated (average) daily new infections based on a detection rate of *δ* = 0.033/day and a lost rate (recovery or death) of non-detected cases of *π* = 0.1/day (D).

Table 1 shows parameter estimates using the whole Italian COVID-19 daily case reporting data from 2020-02-20 to 2020-07-11, with standard errors and 95% confidence intervals. The log-normal distribution based fit recorded the lowest RMSE, and was thus retained for subsequent analyses. The confidence bounds for the parameter *ρ* ($\hat{\rho }=0.32$ with *CI*(*ρ*) = [0.29, 0.35]) indicated that neither the logistic growth model (*ρ* → 0 and *ν* = 1) nor the Bertalanffy-Richards growth model (*ρ* → 0) were appropriate for this dataset. It was noted that *ν* was not significantly different from 1 ($\hat{\nu }=0.85$ with *CI*(*ν*) = [0.70, 1.05]), hence the hyper-logistic model (*ν* = 1) was found to be compatible with the data. The fitted equation (Eq (30) is for *t* ≥ 0

$$\begin{array}{c} {\hat{C}}_{t}=\frac{253124.1}{{\left\{1+{\left[1+0.0242(t-39.3877)\right]}^{-3.1659}\right\}}^{1.1691}} \end{array}$$ (30)

with a coefficient of determination of *R*^2^ = 99.97%. The curves fitted to the new positive cases and the cumulative number of positive cases are shown on Fig 1(A) and 1(B). It can be observed on Fig 1(A) that the peak of new positive cases occurred 29 days after the notification of first case, whereas the maximum likelihood estimate of the theoretical peak time is five days later as shown in Table 2 (${\hat{t}}_{p}=34.10$, *CI*(*t*_*p*_) = [31.94, 36.41] days). The theoretical peak size is on average 5 298 new positive cases (${\hat{\dot{C}}}_{p}=5\;298.96$, $CI\left({\dot{C}}_{p}\right)=\left[4\;609.72,6\;091.25\right]$ new cases) against a maximum of 6 248 observed new positive cases.

**Table 1:** Estimate, standard error (SE) and 95% confidence interval (*CI*_95%_) of Turner’s growth model parameters fitted to the Italian COVID-19 daily case reporting data from 2020-02-20 to 2020-07-11, using the log-normal distribution (RMSE = 514.24, *R*^2^ = 99.97%) and the negative binomial distribution (RMSE = 530.93, *R*^2^ = 99.93%).

Model	Log-normal fit			Negative binomial fit		
parameter	Estimate	SE	*CI*_95%_	Estimate	SE	*CI*_95%_
*K*	253124.1	12623.0	[229554.2, 279114.1]	242952.6	169.6	[242951.2, 242954.0]
*ω*	0.0896	0.0113	[0.0700, 0.1146]	0.0902	0.0098	[0.0729, 0.1117]
*ν*	0.8553	0.0906	[0.6951, 1.0526]	0.8300	0.0771	[0.6918, 0.9959]
*ρ*	0.3159	0.0142	[0.2892, 0.3451]	0.3231	0.0124	[0.2996, 0.3484]
*τ*	39.3877	2.5181	[34.7491, 44.6456]	39.3457	2.2393	[35.1927, 43.9888]
*β*	-4.0229	0.0060	[-4.0348, -4.0111]	-4.0229	0.0060	[-4.0348, -4.0111]
*κ*	0.0076	0.0001	[0.0075, 0.0078]	0.0076	0.0001	[0.0075, 0.0078]
*σ*	0.4332	0.0257	[0.3857, 0.4867]	0.1466	0.0175	[0.1160, 0.1853]

Notes: RMSE = root mean square error; *β* and *κ* define the daily removal rate from detected cases as $\alpha_{t}=\frac{{\text{e}}^{\beta +\kappa t}}{1+{\text{e}}^{\beta +\kappa t}}$; *σ* is the log-normal/negative binomial distribution scale parameter (see the pdf in Eq (27) and pmf in Eq (28)).

From the estimate of the parameter *β* given in Table 1 ($\hat{\beta }=-4.02$ with *CI*(*β*) = [−4.03, −4.01]), it appears that the daily removal rate (recoveries and deaths) averaged ${\hat{\alpha }}_{0}=1.8\%$ in the very early phase of the epidemic (*t* ≈ 0 day). Then, from the estimate of *κ* ($\hat{\kappa }=0.0076$ with *CI*(*κ*) = [0.0075, 0.0076]), it appears that the removal rate increased with time, *i.e*. the probability for an active case to recover or die within a day increased on average by 5.5% over a week. Fig 1(C) displays the active cases and the corresponding fitted curve using the removal probability along with the fitted Eq (30). The active cases were predicted to peak on day 56 (${\hat{t}}_{a}=55.71$ days, *CI*(*t*_*a*_) = [53.87, 57.62] days) to 111 070 active cases (${\hat{A}}_{a}=111\;069.88$ cases, *CI*(*A*_*a*_) = [98 580.39, 125 141.70] cases), whereas the observed peak amounted to 114 683 cases and occurred 58 days after the notification of the first case.

The daily new infections inferred from assuming a constant detection rate (*δ* = 0.033/day) and a constant lost rate (*π* = 0.1/day) is depicted on Fig 1(D). The peak in new infections likely occurred about 28 days (${\hat{t}}_{P}=27.52$ days, *CI*(*t*_*P*_) = [25.62, 29.56] days) after the notification of the first case, and averaged 22 748 new infections (${\hat{\dot{S}}}_{P}=22\;748.38$, $CI\left({\dot{S}}_{P}\right)=\left[19\;726.30,26\;233.44\right]$ new infections) (Table 2). The ratio of the number of infectives to the number of active cases decreased from 44.70 at the first notification day to 11.41 one week later (averaging 22.95, *CI* = [22.01, 23.93] over this period) and to 2.99 at peak time, 22 days later.

**Table 2:** Estimate, standard error (SE) and 95% confidence interval of peak statistics using the COVID-19 daily case reporting data from Italy (2020-02-20 to 2020-07-11).

Quantity	Peak statistic	Estimate	SE	*CI*_95%_	Observed
Detected	Time (day)	34.10	1.14	[31.94, 36.41]	29
	New positive cases	5298.96	376.73	[4609.72, 6091.25]	6248
Actives (isolated)	Time (day)	55.71	0.96	[53.87, 57.62]	58
	Active cases	111069.88	6759.93	[98580.39, 125141.70]	114683
New infections	Time (day)	27.52	1.02	[25.62, 29.56]	-
	New infections	22748.38	1351.44	[19726.30, 26233.44]	-

Notes: - = not available.

### Retrospective fits

The AICs of the retrospective fits of Tuners’s growth model and its special cases to the Italian COVID-19 data of the first two weeks and the first three weeks are presented in Table 3. It can be observed that the best fits correspond to the hyper-logistic growth model for both data of the first two weeks (AIC = 483.03) and data of the first three weeks (AIC = 863.58). Although parsimony indicated the hyper-logistic model fits as the best, the differences Δ_AIC_ in AIC with respect to the full Turner’s growth model fit were mild (|Δ_AIC_| < 2).

**Table 3:** AIC of Turner’s growth model fitted to the Italian COVID-19 daily case reporting data of the first two weeks and the first three weeks from 2020-02-20, with a log-normal distribution for the positive cases.

Dataset	Growth model	Restrictions	NFGMP	AIC	Δ_AIC_
Data of the first two weeks	Full Turner	-	5	484.49	0
	Bertalanffy-Richards	*ρ* → 0	4	504.45	19.97
	Hyper-logistic	*ν* = 1	4	483.03	-1.46
	Logistic	*ν* = 1 and *ρ* → 0	3	530.22	45.73
	Hyper-Gompertz	*ν* → 0 and *ων*^1+*ρ*^ is constant	3	542.92	58.43
	Gompertz	*ν* → 0, *ρ* → 0 and *ων* is constant	2	499.50	15.02
Data of the first three weeks	Full Turner	-	5	864.21	0
	Bertalanffy-Richards	*ρ* → 0	4	901.78	37.57
	Hyper-logistic	*ν* = 1	4	863.58	-0.63
	Logistic	*ν* = 1 and *ρ* → 0	3	945.16	80.95
	Hyper-Gompertz	*ν* → 0 and *ων*^1+*ρ*^ is constant	3	966.71	102.49
	Gompertz	*ν* → 0, *ρ* → 0 and *ων* is constant	2	896.52	32.30

Notes: - = not applicable; NFGMP = Number of free growth parameters; Δ_AIC_ = difference between the AIC of a special growth model fit and the AIC of the full Turner’s growth model fit.

Table 4 shows the estimate of the hyper-logistic growth model parameters for the two shorted datasets. It appears that the estimates of the intrinsic growth parameter *ω* increased slightly with data availability from $\hat{\omega }=0.05$ (*CI*_*ω*_ = [0.04, 0.07]) using the data of the first two weeks, to $\hat{\omega }=0.07$ (*CI*_*ω*_ = [0.06, 0.08]) using the data of the first three weeks and to $\hat{\omega }=0.09$ (*CI*_*ω*_ = [0.07, 0.11]) using the whole dataset from Italy.

**Table 4:** Estimate, standard error (SE) and 95% confidence interval of peak statistics using the COVID-19 daily case reporting data from Italy (2020-02-20 to 2020-07-11).

Model	First two weeks data			First three weeks data		
parameter	Estimate	SE	*CI*_95%_	Estimate	SE	*CI*_95%_
*K*	260124.1	930.9	[258305.9, 261955.1]	260122.6	633.1	[258884.8, 261366.3]
*ω*	0.0518	0.0066	[0.0404, 0.0665]	0.0661	0.0050	[0.0569, 0.0768]
*ρ*	0.3401	0.0183	[0.3061, .3779]	0.3075	0.0121	[0.2846, 0.3322]
*τ*	55.5287	4.3775	[47.5790, 64.8068]	47.7007	2.0405	[43.8645, 51.8724]
*β*	-4.7679	0.2379	[-5.2341, -4.3017]	-3.4678	0.1013	[-3.6663, -3.2693]
*κ*	0.1144	0.0196	[0.0761, 0.1528]	-0.0100	0.0058	[-0.0214, 0.0013]
*σ*	0.2081	0.0393	[0.1437, 0.3014]	0.2165	0.0334	[0.1600, 0.2930]

Notes: *β* and *κ* define the daily removal rate from detected cases as $\alpha_{t}=\frac{{\text{e}}^{\beta +\kappa t}}{1+{\text{e}}^{\beta +\kappa t}}$; *σ* is the log-normal distribution scale parameter (see pdf in Eq (27))

The estimates of the peak time and size from the two shorted datasets are shown in Table 4. The forecast of the peak time from the data of the first two weeks was day 44 (${\hat{t}}_{p}=43.38$, *CI*(*t*_*p*_) = [39.04, 48.22] days) which overestimated the observed peak time (day 29). The estimate from the data of the first three weeks reduced the delay, with ${\hat{t}}_{p}=38.97$ (*CI*(*t*_*p*_) = [36.80, 41.27]) days. The forecast of the peak size from the data of the first two weeks was 3 794 (${\hat{\dot{C}}}_{p}=3\;793.60$, *CI*(*t*_*p*_) = [3 032.63, 4 745.53]) new positive cases (Table 5), which underestimated the observed peak (6248 new positive cases). The forecast from the data of the first three weeks also underestimated the peak but is less biased, with ${\hat{\dot{C}}}_{p}=4\;733.35$ (*CI*(*t*_*p*_) = [4 136.58, 5 416.22]) new positive cases (Table 5).

**Table 5:** Estimate, standard error (SE) and 95% confidence interval (*CI*_95%_) of the parameters of the hyper-logistic growth model fitted using the log-normal distribution to the COVID-19 daily case reporting data from Italy for the first two weeks (RMSE = 92.16, *R*^2^ = 99.68%) and for the first three weeks (RMSE = 224.41, *R*^2^ = 99.87%) from 2020-02-20.

Peak	Data of the first two weeks			Data of the first three weeks		
statistic	Estimate	SE	*CI*_95%_	Estimate	SE	*CI*_95%_
Time (day)	43.38	2.34	[39.04, 48.22]	38.97	1.14	[36.80, 41.27]
New positive cases	3793.60	433.34	[3032.63, 4745.53]	4733.35	325.46	[4136.58, 5416.22]

Notes: RMSE = root mean square error.

## Summary and perspectives

This work proposes the use of a flexible growth model to model case reporting data from an epidemic outbreak with containment measures including at least isolation of individuals tested positive. The generic growth model of [20] offers a flexible framework with the possibility to recover many special growth models such as the common exponential and the logistic growth models, the hyper-logistic, the hyper-Gompertz, the Gompertz and the Bertalanffy-Richards growth models. Since the special models are all nested within the generic model framework, the most appropriate model can be identified using information criteria such as the Akaike’s Information Criterion (AIC), but a likelihood ratio test [28] can also be conducted for models with different number of free parameters. Where additional information can be obtained on the ability to detect infective individuals, the proposed framework allows to include this information so as to infer on the dynamics of the epidemic beyond the identified (positive) cases, without ressorting to mechanistic/compartmental models. Nevertheless, we considered a constant (average) detection rate whereas the detection rate obviously changes over the epidemic course in terms of the detection effort (number of tests, tracing of contact persons).

From our application to the COVID-19 outbreak data in Italy, the hyper-logistic model is the most appropriate model for the dataset. It appears that the modelling approach can predict the dynamics of an epidemic using data from first few days of an outbreak, at least in this example. Indeed, the predicted peak time (and size) for the positive cases (using only the first two/three weeks data) overestimates (and underestimates) the observed peak time (and size). However, the biases can be attributed, for instance, to the increase in the testing effort and isolation (and the subsequent decrease in the growth rate) in Italy where only about 3 762 tests/day were performed in the first three weeks from 2020-02-20, and about 21 248 tests/day were performed in the subsequent three weeks. Our estimate of the ratio of the number of infectives to the number of active cases averaged 22.95 in the first week of the outbreak, within the range [5, 25] obtained by [7] using the SIQR model. Our proposal thus offers a valid alternative to mechanistic models, for instance, the piecewise exponential growth used by [7] within the SIQR model framework on the Italian early outbreak data.

In a very limited data situation, we suggest a further reduction of the number of model parameters to be estimated. Indeed, since the parameter *τ* in the growth model in Eq (2) is a constant of integration determined by the initial conditions of the epidemic, it can be expressed in terms of other parameters and the number of cases *C*_0_ detected at time *t* = 0 as $\tau =\frac{1}{\nu \omega \rho }\{1-{\left[{\left(\frac{K}{C_{0}}\right)}^{\nu }-1\right]}^{-\rho }\}$ for *ρ* ≠ 0 and *τ* = log((*K*/*C*_0_)^*ν*^ − 1)/(*νω*) for *ρ* = 0. Consideration of a procedure where *τ* is not estimated as a free parameter may lead to parsimony, with inference conditional on the number of individuals tested positive at time *t* = 0. Inference on the effective reproduction number and the sensitivity of the epidemic dynamics to the containment measures under the generic growth model framework is considered for future work.
